# Allyl Isothiocyanate (AITC) Triggered Toxicity and *FsYvc1* (a STRPC Family Member) Responded Sense in *Fusarium solani*

**DOI:** 10.3389/fmicb.2020.00870

**Published:** 2020-05-12

**Authors:** Yingbin Li, Yixiang Liu, Zhiping Zhang, Yongsong Cao, Jianqiang Li, Laixin Luo

**Affiliations:** ^1^Department of Plant Pathology, College of Plant Protection, China Agricultural University, Beijing Key Laboratory of Seed Disease Testing and Control, Beijing, China; ^2^Department of Plant Pathology, College of Plant Protection, Yunnan Agricultural University, Kunming, China

**Keywords:** allyl isothiocyanate, filamentous fungi, *Fusarium solani*, *FsYvc1*, sensing

## Abstract

Allyl isothiocyanate (AITC) is a natural product used as a food additive. Due to its strong volatility and broad biological activity, AITC is considered as a bio-fumigant to control soil-borne fungal diseases in agriculture, creating an urgent need for evaluation of the antifungal activity of AITC. Here we study the effect of AITC on *Fusarium solani* growth and explore the molecular mechanisms. The results indicated that AITC causes rapid inhibition of *F*. *solani* after 5 min, hyphal deformity, and electrolyte leakage. A yeast-like vacuolar transient receptor potential channel regulator (*FsYvc1*, a STRPC family member) was identified in *F*. *solani* that seems to play a role in this fungi AITC sensitivity. Genetic evidence suggests the gene *FsYvc1* is involved in *F*. *solani* growth, development, and pathogenicity. Loss of *FsYvc1* resulted in hypersensitivity of *F*. *solani* to AITC and induced reactive oxygen species (ROS) accumulation ∼ 1.3 to 1.45- folds that of the wild type (WT), and no difference responses to CaCl_2_, NaCl, KCl, SDS, and Congo red when compared with WT. In addition, Δ*FsYvc1-17* showed significantly reduced (∼ 1-fold) glutathione-S-transferase (GST) expression compared with the WT without AITC induction. Upon exposure to 4.8 μg/mL AITC for 3 h, the relative expression levels were ∼ 12–30 fold higher in both the WT and Δ*FsYvc1-17*. Nevertheless, no difference in GST expression level was observed between the WT and Δ*FsYvc1-17*. The current study provides novel insights into the toxicity mechanisms of AITC. Considering our results that show the key role of *FsYvc1*, we propose that it could act as a new molecular target for future fungicide development.

## Introduction

The genus *Fusarium* is associated with yield losses in many commercial crops, and can be characterized as a soil-borne fungal pathogen with a broad distribution that is difficult to control (Gao et al., 2019). Associated syndromes include *Fusarium* wilt of strawberry (Henry et al., 2017), damping-off of soybean (Lamichhane et al., 2017), and replanting failure of Sanqi ginseng (Yang et al., 2019) have been reported. Although soil disinfection with fungicides, including dazomet (Basamid^®^), dimethyl disulfide (Paladin^®^), metam sodium (Vapam^®^), methyl-bromide, and chloropicrin (Wang et al., 2006; Watson and Desaeger, 2019), has been used to control this pathogen, most of these are forbidden or restricted within the European Union (Directive 2009/128/CE) and elsewhere due to resistance, environmental, and safety concerns (Pimentel et al., 2007; Gemmill et al., 2013).

Allyl isothiocyanate (AITC), originally isolated from cruciferous plants, has been applied and registered in the medical and food industries (EFSA, 2010; Saladino et al., 2017) for its excellent anti-cancer (Liu et al., 2018) and antimicrobial activities (Nowicki et al., 2016). In 1945, AITC was applied to combat eelworm, and the yields of treated potato plants were 100% above those of controls (Ellenby, 1945). As a “dietary pesticide” (Ames et al., 1990) with broad antimicrobial activity (Angus et al., 1994), AITC has been extensively used in agricultural production to control fungi (Handiseni et al., 2016), bacteria (Charron et al., 2002), nematodes (Ntalli and Caboni, 2012), and weeds (Bangarwa et al., 2012). The proposed action mechanisms of AITC include inhibiting metastasis of cells through suppression of the MAPK pathway (Lai et al., 2014), causing DNA damage through O_2_^–^ generation (Murata et al., 2000), inducing glutathione S-transferase (GST) expression in *Caenorhabditis elegans* (Hasegawa et al., 2010), affecting protein structures by disrupting disulfide bonds in bacteria (Kawakishi and Kaneko, 1987), and killing fungal cells by eliciting an oxidative stress response as in the case of *Alternaria brassicicola* (Calmes et al., 2015).

Transient receptor potential (TRP) channels are polymodal signal detectors that operate in response to a wide array of physical and chemical stimuli (Cordero-Morales et al., 2011). All TRP channels contain at least six transmembrane segments, highly unusual among known ion channel families, and exhibit diverse cation selectivity and specific activation mechanisms (Venkatachalam and Montell, 2007; Chang et al., 2010; Lange et al., 2016). TRP channels can be divided into three subfamilies based on homology: short (S), long (L), and osm (O). Among TRPs, the STRPC family, which includes *Drosophila* TRP and TRPL and the mammalian homologs TRPC1–7, is a group of Ca^2+^-permeable cation channels (Harteneck et al., 2000). In animal and human cell models, TRPA1 plays an important role in regulating channel activity and serves as a biosensor detecting O_2_ (Takahashi et al., 2011); noxious environmental agents (Hinman et al., 2006), such as allicin and diallyl disulfide from garlic and cinnamaldehyde from cinnamon; acrolein, a common air pollutant; and cold or heat stimulation (Bautista et al., 2005, 2006; Hinman et al., 2006). However, a few examples of TRP channels have been identified in fungi and non-land plants (Lange et al., 2016). Fungal TRPs form a distinct subfamily, distinct from the short, osm-like, and long subfamilies previously described in *C. elegans*, *Drosophila melanogaster*, human, and mouse studies (Denis and Cyert, 2002). Three fungal Ca^2+^ channel proteins, CCH1, MID1, and YVC1, were initially characterized in *Saccharomyces cerevisiae*, and the roles of these fungal Ca^2+^ channel genes in growth, development, and pathogenicity have been studied in several species (Kim et al., 2015). The chemical mechanism of AITC is activation of TRPA1 through direct, reversible, and covalent protein modification (Bautista et al., 2005; Hinman et al., 2006; Green and Dong, 2019). These findings provide new insights and concepts for understanding how filamentous fungi sense AITC.

Despite its excellent antimicrobial activity and environmental friendliness, there is limited data on the use of AITC against *Fusarium* and the mechanism requires clarification. The current study used *F*. *solani*, which causes serious continuous cropping obstacles in Sanqi ginseng production in China, as a model to determine the toxicity of AITC using morphology. Furthermore, a TRP homology in *F*. *solani* was characterized and its role in sensing AITC was analyzed using genetics and comparative transcriptome.

## Materials and Methods

### Strains and Chemicals

Total four *F*. *solani* strains (F2, F3, F5, and RR4) were provided by the Key Laboratory of Agro-Biodiversity and Pest Management of the Education Ministry of China, Yunnan Agricultural University, Kunming. Each strain was cultured on PDA and incubated at 25°C for further use. AITC was of analytical grade (ai. ≥ 98%), supplied by Beijing Key Laboratory of Seed Disease Testing and Control, China Agricultural University, Beijing.

### Antifungal Activity *in vitro*

Antifungal activity was assessed using the plate fumigation system with modifications (Troncoso et al., 2005). Briefly, a 5-mm mycelial plug of *F*. *solani* strain F5 was placed in the center of a 9-cm Petri dish containing ∼20 mL PDA. AITC was dissolved in methanol to obtain a stock solution of 96 mg/mL. Assuming the atmospheric volume of Petri dishes was 50 mL and 10 μL AITC stock solution was added to a cotton strip and placed inside the plate, a series of final atmospheric concentrations was attained. The Petri dish was immediately sealed with Parafilm and incubated at 25°C for one week. Control Petri dishes containing 10 μL methanol were included, and four independent biological replicates were used for each treatment. Inhibition of mycelial growth was calculated in percentage terms based on the difference between mycelial growth in the treated and control dishes.

### Observations Using Time-Lapse Photography and Scanning Electron Microscopy

Time-lapse photography experiments were performed with a Nikon ECLIPSE Ti-E inverted microscopy (Japan) at room temperature of 24°C. A 5-mm mycelial plug of *F*. *solani* strain F5 was cultured in the center of a 9-cm Petri dish containing ∼20 mL PDA for 3 days at 25°C. The hyphae first grew without AITC for 20 min, and then AITC was added to the following atmospheric concentrations: 0, 0.3, 0.6, and 1.2 μg/mL at the 20^th^ minute, and the Petri dish was covered. After 20 min, the Petri dish cover was removed. Hyphal tip growth was measured and recorded every 5 min. All images were processed to 8-bit RGB at 2560 × 1920 pixels using automatic white balance, and were acquired at one frame per 20 s over at least 60 min to create movies through automatic export.

The hyphal morphology of treated and untreated fungi was observed using a scanning electron microscopy equipped with the control software HITACHI S-3400N (Hitachi, Tokyo, Japan). All samples were processed consistent with previous descriptions (Li et al., 2015). Briefly, *F*. *solani* strain F5 was cultured on PDA medium at 25°C for 3 days. Then, the fungi were treated with 0.6 μg/mL AITC for 20 min. Mycelial plugs (5 × 5 mm) were collected and fixed with 2.5% (w/v) glutaraldehyde solution at 4°C overnight, dehydrated in an ethanol gradient (30–100% ethanol; v/v), dried with CO_2_ in an HCP-2 critical-point dryer, and sprayed with gold ion sputter (EIKO IB-3).

### Electrolyte Leakage and ROS Detection

Electrolyte leakage analysis was performed using a conductivity meter (DZS-706, Shanghai, China). A 1-mL spore suspension containing 10^6^ spores of *F*. *solani* strain F5 was added to a 50-mL flask containing 30 mL potato dextrose broth (PDB) medium. Each treatment (with or without 64 μg/L AITC) was represented by three flasks incubated at 25°C and 185 rpm for 20 h. Electrolyte leakage was recorded as electric conductivity (μs/cm).

Generation of reactive oxygen species (ROS) by *F*. *solani* strain F5 was measured using a chemical luminescence method (Ito et al., 2007). A fungal mycelial mat (∼ 100 mg) was suspended in 30 mL of PDB. AITC was added to the suspension at a final concentration of 80 μM. After 30 min, the mycelial suspension was filtered with a membrane filter (pore size, 0.22 μm), and the filtrate (1 mL) was mixed with 3.5 mL of 50 mM potassium phosphate buffer (pH 7.8) and 500 μL 1.2 mM luminol in 50 mM potassium phosphate buffer. The reaction was started by the addition of 500 μL of 10 mM potassium ferricyanide. Then, 200 μL was pipetted into the well of a 96-well plate and monitored with a SpectraMax^®^ i3x Multi-Mode Microplate Reader at luminescence 470 nm.

### Exogenous Addition of TRPA1 Inhibitor (HC-030031)

Previous reports have suggested that HC-030031 acts as an effective selective inhibitor against AITC-induced TRPA1 activation *in vitro* (McNamara et al., 2007; Miyake et al., 2016). Here, we added HC-030031 to PDA medium followed by the addition of AITC (atmospheric concentration, 6.0 μg/mL). The method used is described above, with three independent biological replicates for each treatment.

### Characterization of *FsYvc1*

Calcium channel YVC1 of *Saccharomyces cerevisiae* S288C (Accession number: Q12324) was used as a template and a BLASTP search of the *F*. *solani* whole protein sequence (*Nectria haematococca* v2.0) hosted at the Joint Genomics Institute (JGI) was carried out using default parameters. The corresponding gene was defined as *FsYvc1*. The full-length and coding sequences of the *FsYvc1* gene were amplified using the primer pairs A0F+A0R (Supplementary Table S1) from genomic DNA and cDNA, respectively. Transmembrane domains were identified using Phobius (http://phobius.sbc.su.se/) and TMHMM Server v. 2.0 (http://www.cbs.dtu.dk/services/TMHMM/).

### Vector Construction and Transformation

The *FsYvc1* deletion mutant (Δ*FsYvc1*) was constructed and generated as described previously (Zheng et al., 2014). First, the regions 1.0 kb upstream and 1.0 kb downstream were amplified using primer pairs A1+A2 and A3+A4, respectively. The primers HTF/HTR were used to amplify a 3.5 kb fragment encoding the gene replacement cassette *HPH-HSV-tk*, which carries the hygromycin resistance gene, the herpes simplex virus thymidine kinase gene, and the *Aspergillus nidulans* trpC promoter. This cassette was initially amplified from the PtrpChptA-PItk plasmid. Three amplicons were gel purified using the Gel Extraction Kit (Beijing, China) according to the manufacturer’s instructions, and then mixed at a 3:3:3:1 molar ratio with pBluescript SK(-), which resulted in linearization via digestion with *Hin*dIII and *Eco*RI at multiple cloning sites. The In-Fusion cloning procedure was performed using 5 × In-Fusion HD Enyzyme Premix (TaKaRa, Beijing). The deletion vector was confirmed through sequencing. The primers used for *FsYvc1* deletion are listed in Supplementary Table S1.

To prepare protoplasts, 1 × 10^7^ spores of *F*. *solani* strain F5 were added to yeast extract peptone dextrose liquid medium (w/v, 1% peptone, 0.3% yeast extract, and 2% glucose). After 20 h at 120 rpm and 25°C, the young mycelium was filtered through Miracloth (Millipore, United States), washed with 0.7 M NaCl and treated with lysing (5 mg/mL of 0.7 M NaCl; Sigma, United States), driselase (25 mg/mL of 0.7 M NaCl; Sigma), and chitinase (0.05 mg/mL of 0.7 M NaCl; Sigma) enzymes. After 3 h at 85 rpm and 30°C, the enzyme solution was filtered through Miracloth to eliminate mycelial residue. The protoplasts in the filtrate were then washed with 0.7 M NaCl and STC (0.8 M sorbitol, 0.05 M Tris, pH 8.0, and 50 mM CaCl_2_) and resuspended in SPTC (STC with 40% [w/v] PEG6000) buffer (STC: SPTC = 4:1).

For transformation, 10^7^ protoplasts in 200 μL of SPTC buffer and 10 μL (10 μg/μL) of target DNA in 5 μL heparin sodium (5 mg/mL) were mixed and incubated on ice for 30 min; a 1-mL volume of SPTC was mixed with the suspension, which was incubated at room temperature for 20 min. Protoplasts were mixed with 200 mL of regeneration medium (0.1% yeast extract, 0.1% casein hydrolysate, 1.0 M sucrose, and 1.6% granulated agar) when below 40°C, which was poured into 9-cm diameter Petri plates (15 mL per plate) and incubated at 25°C. After 12–24 h, the plates were overlaid with 10 mL of selective agar (1.2% agarose in regeneration medium containing 300 μg/mL of hygromycin B) and incubated further. Transformants were obtained 4 days post-transformation and transferred to fresh PDA with 300 μg/mL hygromycin B. The putative transformants were purified through single-spore isolation and verified using the primer pair A5+A6 and sequencing.

### Comparison of Biological Characters and Sensitivity Assessment

To compare their biological characters, *F*. *solani* strain F5 (WT) and the Δ*FsYvc1-17* were cultured on PDA at 25°C for 4 days. The diameter of each colony was measured, and the number of conidia was determined by counting under a microscope after washing with 10 mL sterile water. The pathogenicity assay was performed *in vitro*, with a 5-mm diameter mycelial plug inoculated onto the root of Sanqi ginseng (3-year-old). Five replicate roots were used for each strain, with two independent biological replicates. Roots were placed in a plastic bag for 48 h to maintain moisture. Disease severity was calculated as the lesion size. Pathogenicity between WT and Δ*FsYvc1-17* were compared using Fisher’s least significant difference (LSD) test.

To assess sensitivity to various stresses, each plate was inoculated with a 5-mm diameter mycelial plug after incubation at 25°C for 4 days on PDA plates with 0.01 M CaCl_2_, 1.0 M NaCl, 1.0 M KCl (osmotic stress agents), 0.05% (w/v) Congo red (cell wall-damaging agent), or 0.05% SDS (w/v, cell membrane-damaging agent). Three replicate plates were used for each treatment.

### The GST Expression Between WT and Δ*FsYvc1-17* in the Response to AITC

Total RNA, including WT and Δ*FsYvc1-17* that were treated and untreated in three biological replicates, were extracted using Eastep^®^ Super kits (Promega, Shanghai) according to the manufacturer’s instructions. Briefly, WT and Δ*FsYvc1-17* were cultured on PDA medium (covered with cellophane) at 25°C for 60 h (∼2.5 cm diameter), and then treated with 4.8 μg/mL AITC for 3 h.

For qRT-PCR, first-strand cDNA was synthesized using a PrimeScript RT Reagent Kit with Genomic DNA Eraser (Takara Bio Inc., Kusatsu, Japan). The expression of GST was analyzed using SYBR Premix Ex Taq (Takara Bio Inc.) and an Applied Biosystems 7500 Fast thermal cycler (ThermoFisher Scientific, United States). The gene-specific primers are listed in Supplementary Table S1. Three biological replicates were used.

### Data Analysis

GraphPad Prism 8.3 software was used for statistical analysis and visualization of datasets. Significant difference analysis was conducted using Fisher’s LSD test in DPS software.

## Results

### Dose and Time-Dependent Responses

**FIGURE 1:**
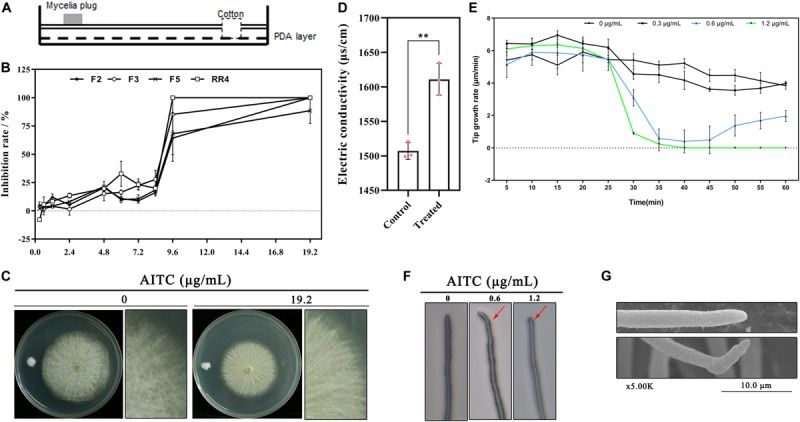
Toxicity of AITC on *F*. *solani* growth *in vitro*: **(A)** Plate fumigation system. **(B)** Colony growth inhibition of different *F. solani* strains (F2, F4, F5, and RR4) at various atmospheric concentrations of AITC (*n* = 4 for each plot, ± standard deviation [SD]). **(C)** AITC displayed strong destructive on *F*. *solani* colony growth at 19.2 μg/mL (colony was incubated on PDA plates at 25°C for three days followed by fumigation with AITC). **(D)** Electrolyte leakage of *F*. *solani* F5 after exposure to 64 μg/mL AITC (*n* = 3 for control/treatment group, *p < 0.05, ± SD). **(E)** Time-lapse photography capturing *F*. *solani* hyphal tip growth at various concentrations of AITC (*n* = 3 for each plot, ±SD). **(F)** Light microscopy observation of *F*. *solani* hyphal tip growth, indicated with red arrow, after exposure to 0.6 to 1.2 μg/mL AITC for 20 min. **(G)** Observation of *F*. *solani* hyphal tip growth after exposure to 0.6 μg/mL AITC for 20 min obtained via scanning electron microscopy.

The antifungal activity of AITC on *F*. *solani* was assessed using the plate fumigation system illustrated in Figure 1A. As shown in Figure 1B, the inhibition ratio was approximately 30% when the atmospheric concentration of AITC was lower than 8.4 μg/mL. At concentrations up to 9.6 μg/mL, all tested strains lost nearly all mycelial growth capacity. As shown in Figures 1C,F. *solani* was incubated on PDA plates at 25°C for three days followed by fumigation with AITC for 24 h at 19.2 μg/mL, resulting in collapse of the mycelial surface, wetness, and loss of its fluffy appearance when compared with untreated *F*. *solani* (0 μg/mL AITC). Further, AITC caused the electric conductivity to increase by ∼ 7%, indicating that the mycelial structure was destroyed (Figure 1D).

Using time-lapse photography, hyphal tip growth was observed and recorded under different atmospheric AITC concentrations. The results were shown in Figure 1E; within 1 h, fungal hyphal tip growth was rapidly declining (beginning at the 25^th^ minute) after exposure to 0.6 μg/mL for 5 min (with AITC added at the 20^th^ minute), and the tip growth rate was 0.6–1.0 μm/min. After removing the Petri dish cover (at the 40^th^ minute), tip growth recovered gradually **(blue line)**, with the maximum growth rate of 2.35 μm/min (at the 60^th^ minute), lower than that of the control (4.07 μm/min). By contrast, fungal hyphal tip growth did not recover by the 60^th^ minute when treated with AITC at 1.2 μg/mL **(green line)**.

The hyphal tip became abnormal after adding 0.6 μg/mL AITC at the 20^th^ minute in most visual fields (Figure 1F and Supplementary Movie S1). After remove of the Petri dish cover at the 40^th^ minute, the tip growth direction changed and the tip stretched following the changed direction. Consistent results were obtained using scanning electron microscopy, including wrinkled and curved hyphae (Figure 1G).

### Characterization of *FsYvc1*

**FIGURE 2:**
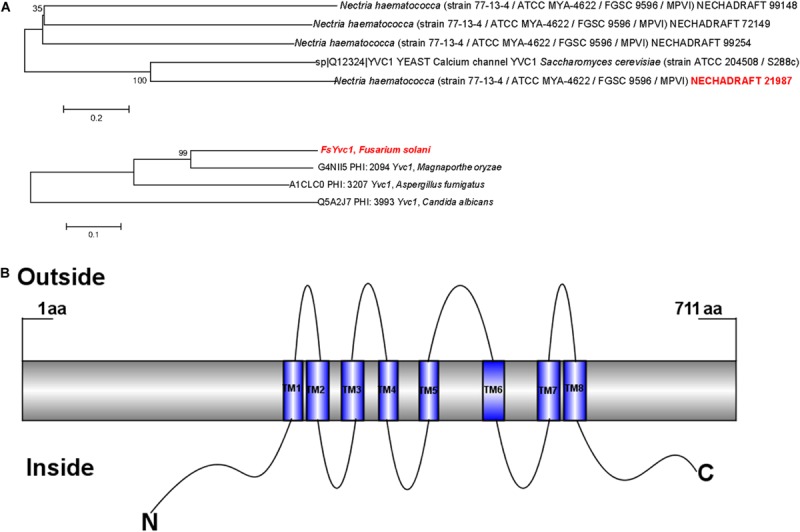
Phylogenetic analysis of FsYvc1 protein sequences and structures: **(A)** BLASTP results for FsYvc1 protein sequences from *Nectria haematococca* (asexual name *F. solani*) and representative fungi. The clade of containing FsYvc1 is highlighted in red font. **(B)** Structure of *FsYvc1*. The full-length amino acid sequence was 711 amino acids, containing eight transmembrane domains (TM1–TM8) with the N- and C-termini both located in the cytoplasm.

The *N*. *haematococca* (asexual name *F. solani*) whole protein sequence was searched by BLASP, and a yeast-like vacuolar transient receptor potential channel regulator Necha 21987 is high homology to YVC1 in *S*. *cerevisiae* and *Magnaporthe oryzae* was obtained (Figure 2A), which belongs to the receptor-activated Ca^2+^-permeable cation channel (KOG Desc. STRPC) family. As shown in Figure 2B, the full length of the amino acid sequence was 711, containing eight transmembrane domains (TM1–TM8) with the N- and C-termini both located in the cytoplasm. This structure is unlike that of STRPC in mammalian cells or *Drosophila*, which have six transmembrane segments (Harteneck et al., 2000).

### Exogenous Addition of TRPA1 Inhibitor

**FIGURE 3:**
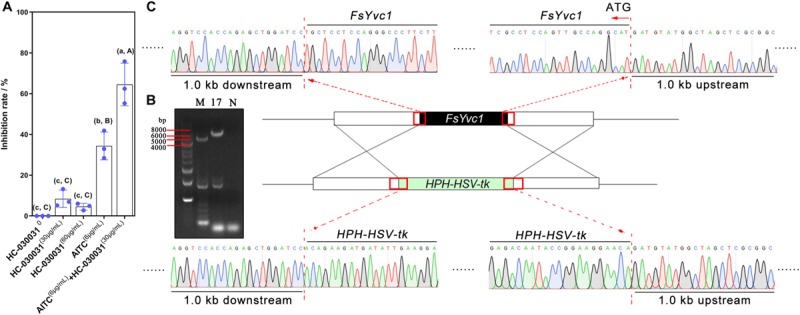
Exogenous addition of a TRPA1 inhibitor, gene deletion, and verification: **(A)** Inhibition rate following exogenous addition of a TRPA1 inhibitor (HC-030031) (*n* = 3 for each treatment, lowercase letters indicate *p* < 0.05, ±SD, uppercase letters *p* < 0.01, ±SD). **(B)** Δ*FsYvc1-17* was screened using PCR (M, marker; N, negative control). **(C)** Sanger sequencing results showing successful substitution of *FsYvc1* with the *HPH*-*HSV*-*tk* cassette.

We hypothesized that when Ca^2+^-permeable cation channels are blocked, *F*. *solani* may fail to sense AITC stimulation and therefore may not establish an effective and timely defense, resulting in hypersensitivity to AITC. Previous reports suggested that HC-030031 acts as an effective selective inhibitor of AITC-induced TRPA1 activation *in vitro* (McNamara et al., 2007). Here, we added HC-030031 to a PDA plate, which was then treated with AITC. As shown in Figure 3A, the inhibition rate was ∼ 4–8% following exogenous addition of HC-030031 alone to PDA at a final concentration of 30 or 60 μg/mL; for treatment with 6.0 μg/mL of AITC alone, the inhibition rate was approximately 38%; combined treatment with AITC and HC-030031, resulted in an inhibition rate of up to 60%. This result indicated that TRP ion channels in *F*. *solani* may play a crucial role in the response to AITC.

### Deletion of *FsYvc1*

To further explore the function of *FsYvc1*, deletion mutants were generated using the gene replacement cassette *HPH-HSV-tk*. As shown in Figure 3B, Δ*FsYvc1-17* was selected and confirmed via PCR amplification and sequencing (Figure 3C).

### Comparison of Biological Characters

**FIGURE 4:**
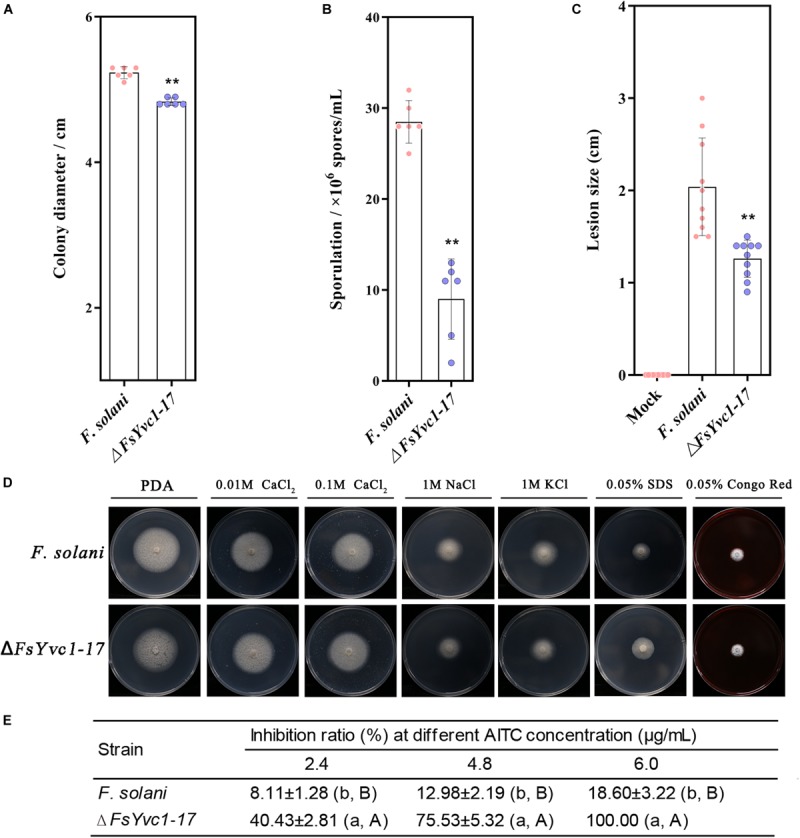
Comparison of biological characters between WT and Δ*FsYvc1-17*: **(A)** Colony growth (*n* = 6 for each plot, **p < 0.01, ±SD). **(B)** Sporulation (*n* = 6 for each plot, **p < 0.01, ±SD). **(C)** Evaluation of pathogenicity by measuring lesion size (*n* = 10 for each plot, **p < 0.01, ±SD). **(D)** Sensitivity to salt cations (calcium, sodium, and potassium), the membrane-damaging agent sodium dodecylsulfate (SDS), and the cell wall-damaging agent Congo red; PDA was used as control. **(E)** Sensitivity to AITC (*n* = 3 for each treatment, lowercase letters *p* < 0.05, ±SD, uppercase letters *p* < 0.01, ±SD).

With the loss of *FsYvc1*, both colony growth and sporulation were significantly reduced compared with the WT (Figures 4A,B). In addition, Δ*FsYvc1-17* exhibited decreased pathogenicity to Sanqi ginseng root *in vitro* (Figure 4C), suggesting *FsYvc1* is involved in hyphal growth, conidial production, and pathogenicity. Further, the sensitivity test showed that Δ*FsYvc1-17* did not exhibit a distinct response to salt cations (calcium, sodium, and potassium), the membrane-damaging agent sodium dodecyl sulfate (SDS), or the cell wall-damaging agent Congo red compared with the WT (Figure 4D). As shown in Figure 4E, Δ*FsYvc1-17* showed hypersensitivity to AITC. When treated with 2.4 μg/mL AITC, the inhibition ratio (IR) of Δ*FsYvc1-17* was about 51%, while it was 10.6% for WT; when treated with 4.8 μg/mL AITC, the IR of Δ*FsYvc1-17* was 97% and was 21.9% for WT; when treated with 6.0 μg/mL AITC, the IR of Δ*FsYvc1-17* was 100% and that of the WT was 36%. These results indicate that Δ*FsYvc1-17* show specificity in responding to external chemical stimuli.

### ROS Accumulation and GST Expression

**FIGURE 5:**
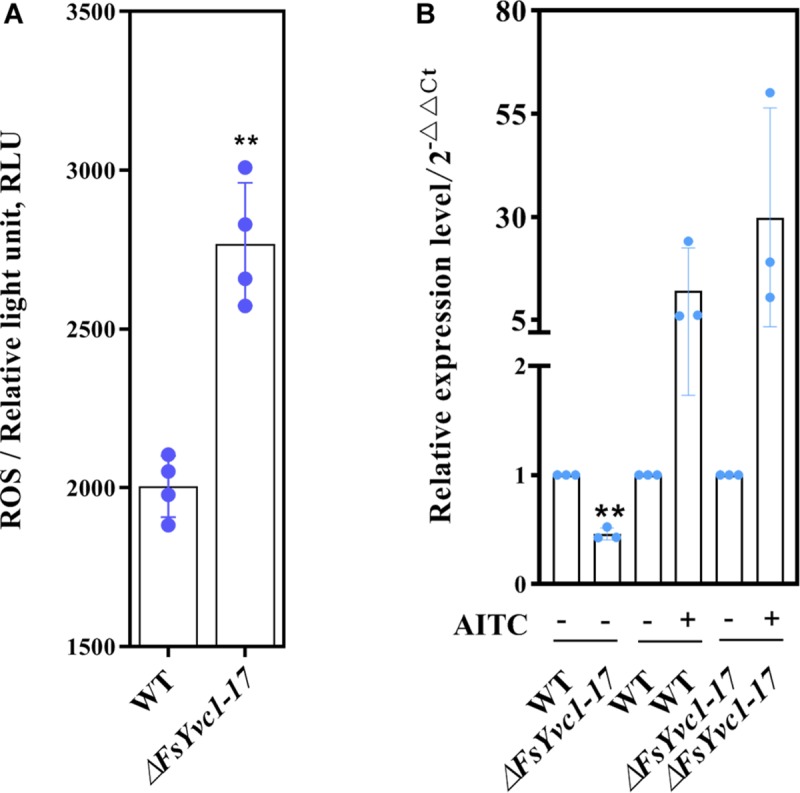
ROS accumulation and GST expression: **(A)** ROS accumulation level (*n* = 4 for each plot, **p < 0.01, ±SD). **(B)** qRT-PCR analysis of GST expression between the WT and Δ*FsYvc1-17* (*n* = 3 for each plot, **p < 0.01, ±SD). “+” and “-” indicate samples treated and untreated with AITC, respectively. The *y*-axis represents normalized fold (2^– ΔΔCT^) compared to untreated (set to 1).

AITC induces the GST, which validates the cancer chemopreventive activity of isothiocyanates (ITCs), related to scavenging ROS in animal tissues (Gupta et al., 2014). Moreover, GST conferred tolerance against AITC-induced oxidative stress in *C. elegans* (Hasegawa et al., 2010). Here, with loss of *FsYvc1*, *F*. *solani* was more susceptible to killing by AITC, presumably due to reduced ability to manage oxidative stress. As shown in Figure 5A, in accordance with previous studies, AITC-induced ROS accumulation in Δ*FsYvc1-17* was ∼ 1.3 to 1.45-folds that of the WT. In GST expression analysis (Figure 5B), the Δ*FsYvc1-17* showed significantly reduced (∼1-fold) GST expression compared with the WT without AITC induction. Upon exposure to 4.8 μg/mL AITC for 3 h, the relative expression levels were ∼ 12–30 fold higher in both the WT and Δ*FsYvc1-17*. Nevertheless, no difference in GST expression level was observed between the WT and Δ*FsYvc1*. Thus, in Δ*FsYvc1-17*, more ROS accumulated compared to the WT, but the ROS scavenging mechanism was not enhanced.

## Discussion

Although previous reports have described the action mechanism of AITC in *Escherichia coli* (Lin et al., 2000), *C. elegans* (Hasegawa et al., 2010), *Sitophilus zeamais* (Zhang et al., 2016), and HepG2 cells (Liu et al., 2018), the mechanism in fungi remains unknown. The present study highlights the toxicity of AITC to filamentous fungi through morphological. Further, we reported a yeast-like vacuolar transient receptor potential channel regulator (*FsYvc1*), and found that loss of *FsYvc1* resulted in hypersensitivity of *F*. *solani* to AITC, suggesting *FsYvc1* should be act as a biosensor in sensing AITC.

**FIGURE 6:**
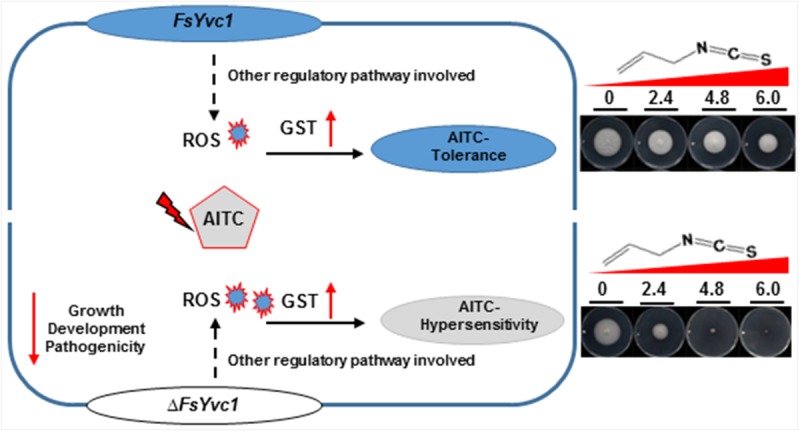
Model illustrating the proposed roles of *FsYvc1* in *F. solani*. The gene *FsYvc1* is involved in *F*. *solani* growth, development, and pathogenicity. *FsYvc1* acts as a security mechanism and is responsible for receiving signals in *F. solani*. Upon triggering by AITC, excess ROS accumulated were induced compared to the WT, but the ROS scavenging mechanism was not enhanced (no difference in GST expression level was observed between the WT and Δ*FsYvc1*) should be the mechanism of the hypersensitive phenotype to AITC.

The mitochondria are likely the main target of AITC, as AITC had a significant effect on the mitochondrial respiratory chain of *S. zeamais in vivo* and *in vitro* (Hua et al., 2014; Zhang et al., 2016). Similarly, ITCs caused to a decreased oxygen consumption rate, intracellular accumulation of ROS, and mitochondrial membrane depolarization in *A*. *brassicicola* (Calmes et al., 2015). Most ITCs can be taken up by cells through passive diffusion. ITCs rapidly conjugate with thiols, particularly glutathione (GSH), and the ITC–GSH conjugate is transported out of the cell as a substrate of multi-drug resistance proteins. The shuttling of ITC–GSH conjugates causes rapid depletion of intracellular GSH, resulting in ROS generation by ITCs (Gupta et al., 2014). In the present study, upon exposure to AITC, the relative expression levels of both the WT and Δ*FsYvc1* were elevated by up to ∼ 12–30 fold. Nevertheless, no difference in GST expression level was observed between the WT and Δ*FsYvc1-17*. Thus, with the loss of *FsYvc1*, accumulation of excess ROS compared to the WT without an enhanced ROS scavenging provides one plausible mechanism of the hypersensitive phenotype (Figure 6).

In traditional antimicrobial assays, such as agar diffusion, AITC is allowed to evaporate into the headspace of the Petri dish, which decreases its antifungal activity (Troncoso et al., 2005). Here, a modified volatilization step of adding AITC to a cotton strip was used for stability and repeatability of antifungal activity evaluation. The results of the present study demonstrated that AITC induced abnormal hyphal growth, electrolyte leakage, destruction of mycelial structure, and ROS accumulation. It is likely that AITC, which shows a broad spectrum of toxicity, has a universal, rapid, and destructive antifungal mechanism.

Cellular responses to physical and chemical stimuli are driven by cell surface receptors, which transmit the signals into cells that cause a response, such as G protein-coupled receptor and TRP channels, both of which act as signal detectors (Cordero-Morales et al., 2011; Gupta et al., 2018). The YVC1 channel, a homolog of the animal transient receptor potential protein 2, resides in the vacuolar membrane and is involved in controlling vacuolar pressure in several filamentous fungi (Chang et al., 2010; Kim et al., 2015). The functions of YVC1 have been characterized in fungi, such as the identification of *Yvc1* (MGG09828.5) as a Ca^2+^ permeable channel involved in fungal development in *Magnaporthe oryzae* (Nguyen et al., 2008), a *yvc1* mutant showed no difference in sensitivity to calcium-depleted, calcium-rich, or alkaline pH conditions in *Candida albicans*. Consistent with prior studies, the present study also demonstrated that *FsYvc1* is related to growth, development, and pathogenicity in *F*. *solani* and does not affect sensitivity to CaCl_2_, NaCl, KCl, SDS, and Congo red. Notably, the *yvc1* mutant of *C. albicans* exhibited reduced stress response capacity and hypersensitive to the membrane-perturbing agent SDS (Yu et al., 2014). In our study, although Δ*FsYvc1-17* showed no difference in sensitivity to SDS, they were hypersensitive to AITC. One possible reason for this result is that AITC acts as a membrane-permeable electrophile (Hinman et al., 2006), and another is evolutionary and genetic differences between *C. albicans* and *F. solani*, as one is a human pathogen and the other is a plant pathogen.

AITC mediated (2.5 mM) redox dysregulation in fungal cells has been described using transcriptomic analysis in *A*. *brassicicola*, with more than one-third of transcripts related to the adaptive response to cellular oxidative stress (Sellam et al., 2007). However, the potential regulatory response may be concealed due to the large dose of AITC. Here, both the WT and Δ*FsYvc1-17* were exposed to 50-fold lower doses of AITC. Notable, GO enrichment analysis in the WT showed results related to heat shock protein binding, coenzyme binding, and cellular metabolic processes, in accordance with previous studies. However, when treated with AITC after loss of *FsYvc1*, enriched GO terms were related to zinc ion binding, transcription factor activity, and regulation of transcription (data not shown). Taken together, these results suggest that *FsYvc1* plays a switch role in sensing AITC so that fungal cells turn “ON” their oxidative stress adaptation mechanisms for AITC tolerance by enhancing the activity of transcription factor and metabolic; however, with the loss of *FsYvc1*, response events mainly occur in the nucleus. This localization may cause failure to sense AITC stimulation and thus prevent timely establishment of an effective defense, resulting in hypersensitivity to AITC. Besides, other possible regulatory pathways in the response to AITC should be discovered in the future.

The present study demonstrates that loss of *FsYvc1* results in AITC hypersensitivity and ROS accumulation along with weak weakly sensitivity to osmotic stress agents, cell wall-damaging agents, and cell membrane-damaging agents. This may be due to cation selectivity and the specific activation mechanisms of TRP (Venkatachalam and Montell, 2007; Chang et al., 2010; Kurganov et al., 2014). In addition, TRP ion channel super family members exhibit six transmembrane segments as a common (Harteneck et al., 2000). However, *FsYvc1* has eight transmembrane domains, explaining why fungal TRPs form a distinct subfamily from TRPs identified in *C. elegans*, *Drosophila melanogaster*, human, or mouse cells (Denis and Cyert, 2002). In present study, although the specific TRPs in *F. solani* that were suppressed by HC-030031 remain unknown, indirect evidence suggested that *FsYvc1* plays a key role in sensing AITC. It is generally accepted that electrophilic agents activate TRPV1 channels through covalent modification of cytosolic cysteine residues, but AITC-induced activation of TRPV1 does not require interaction with cysteine residues, which is largely dependent on S513, a residue involved in capsaicin binding (Gees et al., 2013), however, the possible interactions between AITC and fungi are still unknown and the *FsYvc1* activation and the associated signaling cascade need to elucidate in the further.

Fungal TRPs, including CCH1, MID1, and YVC1, have been initially characterized in *S*. *cerevisiae* (Kim et al., 2015). Here, we focus on the function of YVC1 in *F*. *solani* in response to AITC. In addition, CCH1, a homolog of the a1 subunit of animal voltage-gated Ca^2+^ channels; MID1, a stretch activated channel, constitute a high affinity Ca^2+^ influx system and required for the extracellular Ca^2+^ uptake in response to mating pheromone and also are involved in iron and cold tolerance in yeast (Kim et al., 2015). However, the function of them are still unknown in response to AITC. Notably, other potential TRPs in *F*. *solani* should be further identified, including CE245107_74218, e_gw1.2.1711.1, and MSTRG.9138.2 also induced by AITC in RNA-seq data (data not shown), thus, we think there may be a TRP family in *F*. *solani*, not only including MID1, YVC1, and CCH1 previously reported in *S. cerevisiae*. The transcriptomic data reported begin a new stage in the study and more questions that could be answered in later works. In addition, more candidate transcripts and *F. solani* mutants will be helpful to analysis the mechanism in the future. In addition, patch clamp technology (Neher and Sakmann, 1976), a breakthrough method that has become vital to neuroscience (Reyes, 2019), has been applied in yeast and animal cells to accurately identify ion channel proteins. However, this method is technically difficult to filamentous fungi.

In summary, our morphological, genetic, and transcriptional profiling analyses provide new insights into the possible sensing mechanisms involved in the AITC-triggered response in filamentous fungi. At present, pymetrozine and pyrifluquinazon, two commercially available insecticides, have both been shown to target a TRP ion channel complex unique to insect stretch receptor cells (Nesterov et al., 2015). Considering the distinct families of TRPs in different organisms (nematode, fungus, insect, human, and mouse), this critical function of *FsYvc1* suggests that STRPC could be a potential target for the development of new fungicides.

Since the 1940s, methyl bromide (MB) has been used as a soil fumigant in agricultural production (Lincoln et al., 1942). In particular, resistance, environmental, and safety concerns regarding MB have led to its banning or strict restriction in recent years (Pimentel et al., 2007; Gemmill et al., 2013). AITC represents a potential replacement for MB with high efficiency, low risk, and low molecular weight, and was registered for the control of root knot nematode in tomato in China in 2018 (Ren et al., 2018). Combined with the global trend toward reducing the use of MB, this registration has boosted its commercial prospects in organic production and conventional farming in the future.

## Data Availability Statement

The raw data supporting the conclusions of this article will be made available by the authors, without undue reservation, to any qualified researcher.

## Author Contributions

This research was primarily conducted by YBL, who performed the majority of the experiments, data analysis as well as the preparation of the manuscript. Other authors, including YXL and ZZ performed the antifungal assays and conducted biological characters comparison. JL and LL provided guidance during the experimental design. YC contributed in discussions and in preparation of the manuscript. All the authors listed above read and approved the final version of the manuscript.

## Conflict of Interest

The authors declare that the research was conducted in the absence of any commercial or financial relationships that could be construed as a potential conflict of interest.
