# Supplementary material for: Allyl Isothiocyanate (AITC) Triggered Toxicity and FsYvc1 (a STRPC Family Member) Responded Sense in Fusarium solani
Source: Front Microbiol. 2020 May 12;11:870. doi: 10.3389/fmicb.2020.00870 (PMC7235336; doi:10.3389/fmicb.2020.00870)
Supplement: Supplementary file 2 [file Data_Sheet_1.docx]

**Supporting Information**

**Allyl isothiocyanate (AITC) triggered toxicity and *FsYvc1* (a STRPC family member) responded sense in *Fusarium solani***

Yingbin Li^a^, Yixiang Liu^b^, Zhiping Zhang^a^, Yongsong Cao^a^, Jianqiang Li^a^ and Laixin Luo^a*^

a Department of Plant Pathology, College of Plant Protection, China Agricultural University, Beijing Key Laboratory of Seed Disease Testing and Control, Beijing, China,

b Department of Plant Pathology, College of Plant Protection, Yunnan Agricultural University, Kunming, China

*Corresponding author: Laixin Luo ([luolaixin@cau.edu.cn](mailto:luolaixin@cau.edu.cn))

Number of Pages: 3

Number of Tables: 1

Number of Movie: 1

**Table S1**

Oligonucleotide primers used in this study

| Primer | Sequence(5’-3’)a | Product size (bp) | Relevant characteristics |
| --- | --- | --- | --- |
| A0F | ATGCCTAGCAACTGGAGGCG | 2361 | To amplify the full length of *FsYvc1* |
| A0R | TGCTCCTCCAGGGCCCTTCT | 2133 | To amplify the CDS of *FsYvc1* |
| A1 | cggtatcgataagcttTCAATGCCGCTGCCGG | 1024 | To amplify the 5’ flanking of *FsYvc1* |
| A2 | TCTTCTGTGGATCCAGCTCTGGTGGACC |  |  |
| A3 | AGGAACAGATGTATGGCTAGCTCGCGG | 1023 | To amplify the 3’ flanking of *FsYvc1* |
| A4 | cgggctgcaggaattcTTTTCCAATCTCCAAGATTCTGGAGGAGA |  |  |
| HTF | TGGATCCACAGAAGATGATATTGAAGGAGCATTTTTTGG | 3500 | To amplify the 3.5-kb *HPH-HSV-tk* fragment |
| HTR | CATACATCTGTTCCTTCCGGTATTGTCTCCTTCC |  |  |
| A5 | ATGGCTACCAAATCCATCGGC | 5086 | The PCR products had a size of 5086 bp was wild type and 6207 bp was the putative transformants |
| A6 | CGATGTCGGTGGTTCGTGAAAAG | 6207 |  |
| GS-F2 | GGCTGGTGTTGAAAAGCTCC | 170 | Quantitative real-time PCR primers for analysis of the expression of Glutathione S-transferases (GSTs) gene expression |
| GS-R2 | GGTTCTTGCGTCGCTCAAAG |  |  |
| yq7F | AAGTGAAGAGCGGAAAGGCG | 104 | Quantitative real-time PCR primers for analysis of the expression of *FsYvc1* at different time |
| yq7R | TTCAGGCTCATCAGGGTGC |  |  |
| NADq1F | GCTGGTGACTCCAAGAACGA | 141 | Quantitative real-time PCR primers for analysis of the nicotinamide adenine dinucleotide phosphate (NADPH) gene expression |
| NADq1R | GAACTTGCAGGCAATGTGGG |  |  |

^a^ Lowercase letters indicate the overlap in the primers.

**Movie S1.**

An hour's time-lapse photography to capture the fungal hyphae tip growth process after exposed at different spatial concentration of AITC.
